# The characteristics of risk factors in Chinese young women with acute coronary syndrome

**DOI:** 10.1186/s12872-020-01577-z

**Published:** 2020-06-12

**Authors:** Ruifang Liu, Fangxing Xu, Yujie Zhou, Tongku Liu

**Affiliations:** 1 Department of Cardiology, Beijing Anzhen Hospital, Capital Medical University, Beijing, 100029 People’s Republic of China; 2 The Center of Cardiology, Affiliated Hospital of Beihua University, Jilin 132011 Jilin, People’s Republic of China

**Keywords:** Young women, Coronary artery disease, Acute coronary syndrome, Risk factors

## Abstract

**Background:**

In recent years, the prevalence rate of acute coronary syndrome (ACS) in Chinese young women has been increasing significantly, becoming one of the main causes of death in young females. A matter of constant concern is what is the characteristics and differences in risk factors between young women with ACS and without ACS. This study aimed to investigate the characteristics and difference of risk factors in Chinese young women with ACS and to provide references for ACS prevention and treatment.

**Methods:**

A 1:1 case-control study was conducted to evaluate risk factors of 415 young females with ACS (ACS group) who underwent PCI treatment and 415 young females without ACS (control group) who were hospitalized and confirmed by coronary angiography to exclude coronary heart disease from January 2010 to August 2016. The average age of the cases in groups was respectively (40.77 ± 4.02) and (40.57 ± 4.01) years-old (*P* > 0.05).

**Results:**

The risk factors in ACS group were overweight (64.10%), hypertension (49.88%), hyperlipidemia (40.72%), diabetes (23.37%), depression or anxiety (16.63%), gynecological diseases (16.39%), Hyperuricemia (14.94%), family history of early-onset CHD (14.94%), hyperhomocysteinemia (11.33%), hypothyroidism (9.64%), hypercholesterolemia (8.43%) and high C-reactive protein (7.47%), and were significant difference (*P* < 0.01) compared with that of the control group. The average number of risk factors per case in ACS group was significantly more than that of control groups (*P* < 0.01). Regression analysis showed that hyperlipidemia, hyperhomocysteinemia, overweight (obesity), high CRP, hypertension, hypothyroidism, gynecological diseases, depression or anxiety, cardiac insufficiency, hypercholesterolemia, diabetes, oral contraceptives, family history of early-onset CHD, and autoimmune diseases were independent risk factors (*P* < 0.01). The bivariate correlation analysis between CRP level and age was r = − 0.158 (P < 0.01). The result showed the younger ACS patient is the higher serum CRP.

**Conclusion:**

The independent risk factors of ACS in young women are hyperlipidemia, hyperhomocysteinemia, overweight, high CRP, hypertension, hypothyroidism, gynecological diseases, depression or anxiety, cardiac insufficiency, hypercholesterolemia, diabetes, oral contraceptives, family history of early-onset CHD, and autoimmune diseases. The co-existence of multiple risk factors is the main cause suffering from ACS in young women.

## Background

Coronary heart disease (CHD) is a major disease threatening women’s health and life safety. Epidemiological investigations have shown that acute coronary syndrome (ACS) is one of the leading causes of death in women [1]. Because of the effect of estrogen, the onset age of CHD for women is 10 years later than that of men, and the occurrence of severe clinical events (such as myocardial infarction or sudden death) is 20 years later than that of men [2]。In the last 20 years, the annual mortality rate for young women with CHD has increased by an average of 1.3% [3, 4]。.

In this paper, the age division of peoples is based on the new age segmentation proposed by the World Health Organization in 2017.The age of young people is 44 and under 44 years old, middle-aged people are between 45 and 59 years old, young elderly people are between 60 and 74 years old, elderly people is between 75 and 89 years old, and the old longevity people is above 90 years old. The risk factors of CHD in young women under 44 years old have different characteristics [5]. The characteristics of risk factors in Chinese young women with ACS were reported in the article.

## Methods

### Clinical data and grouping

A retrospective 1:1 case-control study was conducted to evaluate risk factors of 415 young female patients with ACS (ACS group) who underwent percutaneous coronary intervention (PCI) treatment and stenting, and 415 young female cases (control group) who were hospitalized and underwent coronary angiography (CAG) to exclude coronary heart disease from January 2010 to August 2016. The age of the recruited patients was 44 years old or younger with the range from 19 to 44 years old. The average age of the cases in the two groups was respectively (40.77 ± 4.02) years-old and (40.57 ± 4.01) years-old (*P* > 0.05). The matched age conforms to the requirements of the case-control study. The Age, body mass index (BMI), personal history, past medical history, the results of physical and laboratory examination, New York Heart Association (NYHA) cardiac function class, and so on were recorded on admission and during hospitalization.

### Diagnostic criteria

The diagnostic criteria of ACS met the requirements described in the American College of Cardiology / American Heart Association and European Society of Cardiology guidelines for the management of patients with non-ST-elevation and ST-segment elevation ACS [5–8]. The patients with ACS were at least one coronary artery stenosis of 75% or more in vessel diameter, or complete occlusion of infarction relative artery (IRA) confirmed by coronary angiography. The completely blocked IRA was defined as the blood flow of the Thrombolysis in Myocardial Infarction (TIMI) 0–1 grade. In the ACS group, there were 304 cases (73.25%) with unstable angina pectoris, 28 cases (6.75%) with non-ST-segment elevation acute myocardial infarction (NSTEMI), and 83 cases (20.00%) with acute ST-segment elevation myocardial infarction (STEMI). The cases in the control group were all patients without typical chest pain and received coronary angiography examination, which showed coronary artery was no stenosis or diameter stenosis of less than 50%. The classification of cardiac function was diagnosed according to NYHA classification criteria [9]. The diagnostic criteria of renal insufficiency meet the standard of the National Kidney Foundation K/DOQI Clinical Practice Guidelines [10]. The patients with renal insufficiency were all in the compensatory period.

### The judging criteria for risk factors

(1) Body-mass index (BMI) is equal to weight (kg)/height (m^2^) squared. Overweight is defined as BMI ≥ 24 (WHO’s recommendation for BMI normal range is (18.5–23.0 kg/m2) for Asians and overweight is BMI ≥ 24) [11]. (2) PCI history refers to that the patients received PCI treatment at any time before admission. (3) Family history of early-onset CHD refers to that the patients suffered from CHD or died of CHD or of unknown causes before the age of 50. (4) Smoker refers to that the patient was smoking within the past week or smoking on admission. (5) The patients who had suffered from hypertension in the past and had been treated with antihypertensive drugs were recorded as having hypertension. (6) The hyperlipidemia was defined as that the patient had suffered from hyperlipidemia, was taking lipid-lowering drugs, or that anyone of total cholesterol (TC), triglyceride (TG) and low-density lipoprotein cholesterol (LDL) of fasting venous plasma had exceeded the normal reference limit at the first time on admission. (7) TC > 6.0 mmol/L at the first time on admission was recorded as hypercholesterolemia.(8) Plasma homocysteine (Hcy) >15umol/L was recorded as hyperhomocysteinemia. (9)C-reactive protein (CRP) > 10 mg/L was recorded as high CRP; (10) Hemoglobin (HB) < 110 g/L was recorded as anemia. (11) It was recorded as diabetes that the patient has been diagnosed with diabetes before admission, or is receiving hypoglycemic drug treatment, or is the fasting venous blood glucose value ≥7.0 mmol /L on admission. (12) It was recorded as menopausal that the patient had not menstruated for 3 months or more. (13) The patients with cardiac insufficiency refers to the these whose the New York Heart Association cardiac function (NYHA) classification was 2 to 4 class, or the left ventricular ejection fraction measured by echocardiography was less than 50% and the diastolic left ventricular inner diameter was greater than 53 mm. (14) The serum creatinine (SCr) level > 133 mmol/L on admission or evaluated Glomerular Filtration Rate (eGFR) < 90 ml/min/1.73m^2^ (evaluated by using CockcroftGault equation from serum creatinine levels) [12] was recorded as chronic renal insufficiency (In the study eGFR of the patients with chronic renal insufficiency were 60-89 ml/min/1.73m^2^). (15) The uric acid value >375umol/L on admission was recorded as hyperuricemia. (16) It was recorded as renal artery stenosis that the patients with high blood pressure and with one or bilateral renal artery blood vessel diameter stenosis≥50% by renal artery color ultrasound or by CT renal artery vascular imaging or by invasive renal artery angiography or by undergoing renal artery stent implantation.(17) It was recorded as hypothyroidism that patient suffered from hypothyroidism and was taking thyroid hormone therapy or plasma thyroxine level was lower than the normal level, and thyrotrophin was elevated at the same time.(18) It was recorded as an autoimmune disease that patient suffered from autoimmune disease in the past week and was taking related drugs treatment.(19) If the patients had been diagnosed with depression or anxiety previously and were taking antidepressant medication, these patients were recorded as having depression or anxiety. (20) It was recorded as gynecological diseases that the patient had been diagnosed as gynecological diseases on admission or ago, and was treated to be taking medication.(21) The patients admitted to be on oral contraceptives were recorded as being on oral contraceptives.

### Exclusion criteria

The patients with the following conditions shall not be included in the study: (1) The acute myocardial infarction caused by coronary artery bypass graft (bridging vessel) lesions; (2) The acute myocardial infarction because of coronary aneurysm; (3) Patients with uncontrolled local or systemic infectious diseases; (4) Patients with malignant tumors; (5) Patients with hematologic disorders, such as hemophilia, thrombocytopenia, moderate or severe anemia (HB ≤ 80 g/L or HB ≤ 60 g/L), and (6) STEMI patients who refuse PCI.

### CAG and PCI methods

The Femoral artery or radial artery approach was adopted for CAG and PCI. CAG was performed according to conventional cardiac interventional techniques. CAG video was carefully read by two qualified interventional cardiologists to determine that the culprit coronary vessels were suitable for PCI. After the patient signed the informed consent, the heparin sodium was supplemented to 100 units/kg, and PCI was performed as a routine method.

### Perioperative medication during PCI

4000u of heparin sodium was routinely injected by intravenous bolus during CAG. Loading dose of 300 mg of aspirin and 600 mg of clopidogrel hydrogen sulfate were routinely administered orally before or after PCI”. After CAG was performed the heparin sodium was supplemented to 100u/Kg for patients with ACS-PCI. Heparin sodium 1000u was supplemented by intravenously when the PCI procedure was lasting for every 1 h. The whole blood activation coagulation time (ACT) ≥ 350 s was maintained until PCI was completed.

### Success criteria of PCI

Successful PCI was defined as that after drug eluting stent (DES) implantation, the residual stenosis of the target coronary vessel segment was less than 10%, and the forward blood flow in the target lesion vessel reached TIMI 3 grade [13], and no death and no acute myocardial infarction occurred during PCI. The subjects in the ACS group were all successful PCI.

### Statistical analyses

The sample size of this study was calculated by using Power Analysis and Sample Size software (PASS, v 11.0.10, developed by NCSS, LLC, Kaysville, Utah, USA). According to the statistics principle and the epidemiology research theory in the multi-factor analysis, body mass index (BMI) was used as the exposure index to calculate sample size. The following statistical parameters were prepared by referring to the relevant literature and were input into the computer PASS software. After setting all statistical parameters, the sample size was got from PASS automatic calculation. The parameters were shown as follows: power (1-β) is equal to 0.80, β is equal to 0.20, alpha (significance level) is equal to 0.05, M (number of controls per case) is equal to 1, P0 (probability that a control is exposed) is equal to 0.10 and OR value is equal to 2.0. The sample size of 369 cases (that is at least sample size) was got form output result. Since it was a 1:1 case-control study, the sample sizes of the two groups were equal, that is 369 + 369 = 738 cases. In the period of case collection (from January 2010 to August 2016) there were 434 patients with ACS - PCI who initially met the inclusion criteria. Among them 19 patients were excluded (5 patients with acute myocardial infarction and with coronary aneurysm, 8 patients with moderate or severe anemia (HB ≤ 80 g/L or HB ≤ 60 g/L) because of functional uterine bleeding, and 6 STEMI patients who refuse PCI because the chest pain had eased on admission). Final total 415 of 434 cases with ACS were selected consecutively as ACS group. Within the same recruitment time, 415 cases without the coronary artery disease confirmed by CAG examination were selected as control group. The final number of recruited patients in each group, 415 cases, were much more than the minimum sample size, 369 cases in each group.

The data statistical analyses were performed using the Statistical Package for Social Sciences software (SPSS, version 20.0, SPSS Inc. Chicago, IL, USA). All data of cases were input into the computer software database. The continuous variables with normal distributions were expressed as mean ± standard deviation. The comparisons between groups were performed using the independent Student’s t-test. The counting data were expressed as a percentage (%), and the chi-square(X^2^) test was used for comparison between groups. Risk factors were analyzed by Logistic regression. The relationship between CRP level and age was analyzed by using Bivariate Correlation Analysis. The test level was set as a double-tail test a = 0.05. *P* < 0.05 was statistically significant, and *P* < 0.01 was statistically very significant.

## Results

### Comparative analysis of age and risk factors between the two groups

The age of patients in the ACS group and in the control group was respectively (40.77 ± 4.02) years-old and (40.57 ± 4.01) years-old, and was no significant difference compared between two groups (*P* > 0.05). There was no significant difference in age distribution between the two groups (P > 0.05) (see Table 1)。The distribution strength of risk factors in ACS group was overweight (64.10%), hypertension (49.88%), hyperlipidemia (40.72%), diabetes (23.37%), depression or anxiety disorder (16.63%), gynecological diseases (16.39%), hyperuricemia (14.94%), family history of early-onset CHD (14.94%), high homocysteine (11.33%), low thyroid function (9.64%), high cholesterol (8.43%), high C - reactive protein (7.47%), and was significant difference compared between the two groups(*P* < 0.01). Other risk factors account for a smaller proportion (see Table 2). The baseline data of coronary angiographic characteristics and stents implantation in ACS group was showed in Additional files: Supplemental data Table 1.

**Table 1:** The age distribution between the two groups

Age (years old)	< 30	30–39	40–44	mean ± standard deviation
ACS group, n (%)	12 (2.89)	90 (21.69)	313 (75.24)	40.77 ± 4.02
Control group, n (%)	14 (3.37)	90 (21.69)	311 (74.94)	40.57 ± 4.01

Compared with the number of cases distribution in two groups, X^2^ = 0.160, *P* = 0.923. Compared with the mean number of patients in the two groups, t = 0.725, *p* = 0.468

**Table 2:** Comparison of risk factors between ACS group (*n* = 415) and control group (*n* = 415)

	ACS group	Control group	X^2^ value	*P* value
Overweight n (%)	266 (64.10)	69 (16.63)	194.250	0.000
Hypertension n (%)	207 (49.88)	46 (11.08)	147.378	0.000
Hyperlipidemia n (%)	169 (40.72)	13 (3.13)	171.27	0.000
Diabetes n (%)	97 (23.37)	17 (4.09)	65.079	0.000
Depression or anxiety n (%)	69 (16.63)	10 (2.41)	48.698	0.000
Gynecological diseases n (%)	68 (16.39)	9 (2.17)	49.813	0.000
Hyperuricemia n (%)	62 (14.94)	27 (6.51)	15.417	0.000
Family history of CHD n (%)	62 (14.94)	9 (2.17)	43.264	0.000
Hyperhomocysteinemia n (%)	47 (11.33)	4 (0.96)	38.628	0.000
Hypothyroidism n (%)	40 (9.64)	4 (0.96)	31.103	0.000
Hypercholesterolemia n (%)	35 (8.43)	7 (1.69)	19.662	0.000
High CRP n (%)	31 (7.47)	4 (0.96)	38.628	0.000
Anemia n (%)	33 (7.95)	15 (3.61)	7.164	0.011
Cardiac insufficiency n (%)	31 (7.47)	1 (0.24)	29.253	0.000
Smoking n (%)	27 (6.51)	12 (2.89)	6.054	0.021
History of PCI n (%)	25 (6.02)	0 (0.00)	25.776	0.000
Autoimmune diseases n (%)	21 (5.06)	4 (0.96)	11.919	0.001
Postmenopausal n (%)	18 (4.34)	4 (0.96)	9.152	0.004
Oral contraceptive n (%)	15 (3.61)	2 (0.48)	10.149	0.002
Renal insufficiency n (%)	10 (2.41)	0 (0.00)	10.122	0.002
Renal artery stenosis n (%)	4 (0.96)	0 (0.00)	4.019	0.124

*CHD* coronary heart disease, *CRP* C-Reactive Protein, *Family history of CHD* Family history of early onset CHD, *PCI* percutaneous coronary intervention

### The number distribution of combined risk factors in the two groups (see Table 3)

Table 3 shows the number of combined risk factors per case. Zero is no risk factor, and 1–7 is the number of combined risk factors. As shown in Table 3, in the control group, 77.35% of individuals had no risk factors, only 22.65% of cases had 1–3 risk factors. Among the patients in the ACS group, only 7.95% had no risk factors, 54.70% had 2 to 3 risk factors, 85.79% had 1 to 4 risk factors, and 92.05% had 1 to 7 risk factors. The number of combined risk factors was statistically significant compared between the two groups (*P* < 0.01).

**Table 3:** The distribution of risk factors number in ACS group (*n* = 415) and control group (*n* = 415)

No. of risk factors	ACS group	Control group
0, n (%)	33 (7.95)	321 (77.35)
1, n (%)	88 (21.21)	72 (17.35)
2, n (%)	126 (30.36)	21 (5.06)
3, n (%)	101 (24.34)	1 (0.24)
4, n (%)	41 (9.88)	0 (0.00)
5, n (%)	20 (4.82)	0 (0.00)
6, n (%)	5 (1.20)	0 (0.00)
7, n (%)	1 (0.24)	0 (0.00)

X^2^ = 475.944, *P* = 0.000

### Combined risk factors of overweight cases

Overweight is the first risk factor of ACS for young women. In the ACS group, 266 (64.10%) patients were overweight (obese). The proportion of overweight combined risk factors was shown in Table 4. As can be seen from Table 4, among 266 overweight patients in the ACS group there was no alone overweight (obese), while 98.13% of the overweight cases were combined with 1 to 5 other risk factors, and 1.50% cases with six risk factors and 0.38% case with seven risk factors. In the control group, there were only 69 cases with overweight. Among 69 cases, 6 (8.70%) had no combination of any risk factors, 89.86% had a combination of 1 to 2 risk factors, and 1.45% had combination of 3 risk factors. There was a statistically significant difference in the distribution of combined risk factors of overweight cases compared between the two groups (*P* < 0.05). The proportion distribution of the patients with different degrees of obesity in two groups was showed in Additional files: Supplemental data Table 2.

**Table 4:** Distribution of combined risk factors of overweight cases in ACS group (*n* = 266) and control group (*n* = 69)

Combined risk factors	ACS group	Control group
0, n (%)	0 (0.00)	6 (8.70)
1, n (%)	47 (17.67)	43 (62.32)
2, n (%)	84 (31.58)	19 (27.54)
3, n (%)	82 (30.83)	1 (1.45)
4, n (%)	30 (11.28)	0 (0.00)
5, n (%)	18 (6.77)	0 (0.00)
6, n (%)	4 (1.50)	0 (0.00)
7, n (%)	1 (0.38)	0 (0.00)

X^2^ = 84.871, *P* = 0.000

### Comparison of blood biomarkers in different group (see Table 5)

It can be seen from Table 5 that there was no significant difference in HB (g/L), TC (mmol/L), and Uric (mmol/L) compared between two groups (*P* > 0.05). Serum LDL (mmol/L), TG (mmol/L) and TC/HDL ratio, SCr (umol/L), CRP (mg/L) and Hcy (ummol/L) in ACS group were significantly higher than that in control group (*P* < 0.05). However, the HDL/LDL ratio (0.53 ± 0.19) in the control group was significantly higher than that (0.46 ± 0.18) in the ACS group (*P* < 0.01). The TC/HDL ratio (4.19 ± 1.52) in the ACS group was significantly higher than that (3.58 ± 0.93) in the control group (P < 0.01).

**Table 5:** Comparison of biomarkers in different groups (mean ± standard deviation)

Biomarkers	ACS group	Control group	X^2^	*P* value
HB(g/L)	127.85 ± 15.20	129.39 ± 12.10	1.613	0.107
LDL (mmol/L)	2.64 ± 1.11	2.46 ± 0.63	2.939	0.003
HDL (mmol/L)	1.09 ± 0.26	1.24 ± 0.35	7.059	0.000
HDL/LDL	0.46 ± 0.18	0.53 ± 0.19	4.892	0.000
TG (mmol/L)	1.81 ± 1.57	1.38 ± 0.50	5.239	0.000
TC (mmol/L)	4.36 ± 1.33	4.24 ± 0.92	1.432	0.153
TC/HDL	4.19 ± 1.52	3.58 ± 0.93	6.978	0.000
SCr (umol/L)	63.40 ± 19.32	59.22 ± 11.12	3.755	0.000
Uric (umol/L)	285.30 ± 83.73	277.15 ± 66.48	1.552	0.121
CRP (mg/L)	3.40 ± 5.98	1.24 ± 1.46	7.124	0.000
Hcy (ummol/L)	10.30 ± 6.04	8.46 ± 2.45	5.732	0.000

*CRP* C-reactive protein*, HB* Hemoglobin*, Hcy* Homocysteine*, HDL* high density lipoprotein cholesterol, *LDL* low density lipoprotein cholesterol, *SCr* serum creatinine, *TC* total cholesterol, *TG* triglyceride, *Uric* Uric acid

### Logistic regression analysis of ACS risk factors

ACS as the dependent variable and the overweight, hypertension, hyperlipidemia, diabetes, depression or anxiety, gynecological diseases, high uric acid, family history of early-onset CHD, low thyroid function, chronic cardiac insufficiency, smoking, history of PCI therapy, autoimmune diseases, menopause, birth control pills, chronic renal insufficiency, renal artery stenosis as independent variables were generated into the equation to be analysis (see Table 6). The confidence interval of 95% of relative risk (RR) was respectively hyperlipidemia (12.21—46.94), high homocysteine (Hcy, 5.32—43.76), overweight (4.95—11.07), high CRP (3.22—35.82), high blood pressure (2.80—6.87), low thyroid function (2.79—31.13), gynecological diseases (2.59—14.23), depression or anxiety (2.40—12.71), cardiac insufficiency (2.25—155.03), Hypercholesterolemia (2.23—12.21), diabetes (2.14—7.96), oral contraceptive (2.08—65.58), family history of early-onset CHD (1.66—9.86), and autoimmune diseases (1.60—22.75) (*P* < 0.01). (Detailed regression parameters are shown in Additional file: Supplementary data Table 3.)

**Table 6:** Multivariate Logistic regression analysis of ACS risk factors

Risk factors	RR	95%CI	*P* value
Hyperlipidemia	23.940	12.209—46.943	0.000
Homocysteinemia	15.260	5.321—43.763	0.000
Overweight	7.403	4.950—11.070	0.000
High CRP	10.740	3.220—35.817	0.000
Hypertension	4.384	2.799—6.869	0.000
Hypothyroidism	9.323	2.792—31.133	0.000
Gynecological disease	6.609	2.588—14.232	0.000
Depression or anxiety	5.516	2.395—12.705	0.000
Cardiac insufficiency	18.692	2.254—155.030	0.000
Hypercholesterolemia	5.287	2.228—12.214	0.000
Diabetes	4.125	2.138—7.958	0.000
Oral contraceptive	11.684	2.082—65.581	0.005
Family history of CHD	4.043	1.657—9.863	0.002
Autoimmune diseases	6.023	1.595—22.746	0.008

*CRP* C-reactive protein, *Family history of CHD* Family history of early onset coronary heart disease, *RR* relative risk, *95%C.I* 95% confidence interval

### Bivariate correlation analysis between CRP level and age

The mean age of 830 cases was (40.67 ± 4.019) years old. The mean serum CRP of them was (2.32 + 4.48) mg/L. A Bivariate Correlation Analysis of CRP level and age yielded a Pearson Correlation of − 0.127 and a *P* value of 0.000 for the double-tailed test. This result indicates that the CRP level is negatively correlated with age; that is, the younger patient is the higher CRP level. This discovery indicates CRP plays a role in the pathogenesis of young female patients with ACS (See Additional file: Supplemental data Table 4).

## Discussion

### The intensity of risk factors in young women with ACS

Epidemiological studies have shown that hypertension, hyperlipidemia, smoking, and diabetes are the four major risk factors for coronary heart disease [14, 15]. The results of this study showed that the age of young women was (40.77 ± 4.02) years-old in ACS group and (40.57 ± 4.01) years-old in the control group, and was no significant difference (*P* > 0.05) compared between two groups. The subjects in the control group received coronary angiography examination and were proved to be not the coronary artery lesion or to have only mild atherosclerotic plaque, and stenosis with less than 50% of coronary artery lumen diameter. The proportion of combined risk factors in the control group was significantly lower than that in the ACS group (*P* < 0.05). The risk factors in ACS group was respectively overweight (64.10%), hypertension (49.88%), hyperlipidemia (40.72%), diabetes (23.37%), depression or anxiety disorder (16.63%), gynecological diseases (16.39%), hyperuricemia (14.94%), family history of early- onset coronary heart disease (14.94%), hyperhomocysteinemia (11.33%), low thyroid function (9.46%), high cholesterol (8.43%), high CRP (7.47%). Other risk factors account for a smaller proportion (see Table 2). However, except for renal artery stenosis, there were statistically significant differences in the distribution of risk factors compared between groups(*P* < 0.01). These results are consistent with those of the Framingham Heart Study [16, 17]. The result of Framingham Heart Study showed the relative risk of coronary heart disease in obese women increased by 64% and was statistically significant differences compared with a 46% increase in men. The relationship between obesity and cardiovascular disease is becoming more and more clear [18]. The regression analysis in this study shows that the relative risk in obese (overweight) women with ACS was more than 400% of those in normal young women without ACS(*P* < 0.01). The results of this study showed that 64.10% of cases with overweight in the ACS group were significantly higher than those (16.63%) in the control group (P < 0.01). Of the 266 overweight (obese) patients in the ACS group, 98.13% were overweight with one to five risk factors (P < 0.01). This result showed that the combination of multiple risk factors is one of the main causes of overweight cases with ACS. In addition to the four traditional risk factors of hypertension, hyperlipidemia, smoking, and diabetes, the regression analysis results showed that in young women, hyperhomocysteinemia, overweight /obesity, high CRP, hypothyroidism, gynecological diseases, depression or anxiety, cardiac insufficiency, and oral contraceptives, family history of early-onset CHD and autoimmune diseases are independent risk factors. This is the main characteristic of risk factors in young women with ACS [2]. In the ACS group, the distribution proportion of these risk factors was significantly higher than that in the control group (*P* < 0.01). Among the ACS patients in this study, 54.70% of cases were combination with 2 to 3 risk factors, and 85.79% were combination with 1 to 4 risk factors. There were statistically significant differences in the number of combined risk factors compared between the two groups (*P* < 0.01). This result indicates that the co-existence of multiple risk factors is the main cause for the occurrence of ACS in young women. It has been reported that gynecological diseases combined with cardiovascular disease (such as hypertension) and diabetes increase the risk of ischemic heart disease by two times [19]. The patients who smoke and also take oral contraceptives have a seven-fold increased risk of developing arteriosclerotic cardiovascular disease [20]。The results of this study also showed that the smoking rate of ACS patients was significantly higher than that of the control group (*P* < 0.05). Hyperuricemia is an independent risk factor in women with CHD, but not in men [21]。Estrogen has a protective effect on the heart [22, 23]. After menopause, estrogen secretion gradually decreases in women, which causes metabolic disorders and leads to increased blood lipids and increased blood viscosity, thus leading to atherosclerosis and significantly increasing the incidence of coronary heart disease [24]. So menopause is a unique risk factor for coronary heart disease in women [25].

### The role of lipids, creatinine, uric acid, homocysteine and inflammatory reactions on the pathogenesis of ACS

The results of this study showed that the serum levels of LDL, TG, creatinine (Crea), CRP, and Hcy in the ACS group were significantly higher than those in the control group (*P* < 0.01). This showed that these factors are involved in the occurrence and development of ACS in young women [26]. The proportion of hyperlipidemia (40.72%) in the ACS group was significantly higher than that (3.13%) in the control group (*P* < 0.01). Hyperuricemia (14.94%) in the ACS group was significantly higher than that (6.51%) in the control group (P < 0.01). The HDL/LDL ratio (0.53 ± 0.19) in the control group was significantly higher than that (0.46 ± 0.18) in the ACS group (P < 0.01). The TC/HDL ratio (4.19 ± 1.52) in the ACS group was significantly higher than that (3.58 ± 0.93) in the control group (*P* < 0.01). This indicates that lipids and uric acid are involved in the pathogenesis of ACS in young women [27, 28]. Hcy has been proven to be a risk factor for coronary heart disease. Hcy level (10.30 ± 6.04umol/L) in the ACS group was significantly higher than that (8.46 ± 2.45umol/L) in the control group, with a statistically significant difference (*P* < 0.01) compared between two groups. This indicates that Hcy is also a risk factor suffering from ACS in young women.

The relationship between CRP and ACS is complex. CRP promotes the formation of unstable plaques of coronary atherosclerosis, and triggers the rupture of vulnerable plaques, cause thrombosis in coronary arteries and lead to the occurrence of ACS and acute myocardial infarction (AMI). CRP can reduce the stability of nitric oxide synthase mRNA in endothelial cells, which leads to the decrease of the expression of nitric oxide synthase protein and to inhibits endothelial cells to produce nitric oxide. Nitric oxide has the role of maintaining the elasticity of blood vessel, and dilating the blood vessels, being against vasoconstriction caused by endothelin and angiotensin II, and promoting the formation of blood vessels. CRP may also increase the expression of endothelial cell adhesion molecules and enzymatically bind to modified low-density lipoprotein, which promote monocyte aggregation into the atheromatous plaque to cause plaque instability. AMI necrosis substance stimulates the production of CRP. Therefore, the increase in CRP is proportional to the number of myocardial cells necrosis. CRP rises to the peak value in 2 days after the onset of AMI, and then drops gradually. The finding suggests that there is a causal relationship between CRP and the onset of ACS, and that CRP is an important risk factor for the onset of ACS in young women. This study showed that the average CRP (3.40 ± 5.98 mg/L) in the ACS group was significantly higher than that (1.24 ± 1.46 mg/L) in control group(*P* < 0.01). A bivariate correlation analysis of CRP level and age yielded a Pearson Correlation of − 0.129 and a *P* value of 0.000 as the double-tail test. This result showed that CRP level is negatively correlated with age; that is, the younger female is the higher CRP level, indicating the role of CRP on the occurrence and development of ACS in young females. Previous studies have shown that high sensitivity C-reactive protein (hs-CRP) elevation can predict future adverse cardiac events [29]. Anti-inflammatory drugs, such as colchicine, methotrexate, and IL-1β inhibitor canakinumab, can reduce the inflammatory response and significantly reduce the risk of cardiovascular events. The result confirms that the inflammatory response is involved in the occurrence and development of ACS.

### The limitations of this study

This study has some limitations. The effect of the duration of exposure risk factors on the occurrence of ACS is still unclear. It is not clear how long the risk factors exposed last for before they become effective. Such unanswered questions are definitely requiring a further study to address.

## Conclusion

Comprehensive above, can be concluded that: (1) the young female with ACS is to have risk factors of complex. Hyperlipidemia, high homocysteine, overweight, high CRP, hypertension, hypothyroidism, gynecological diseases, depression or anxiety, cardiac insufficiency, hypercholesterolemia, diabetes, oral contraceptive, family history of early -onset CHD, autoimmune diseases are independent risk factors for young women with ACS; (2) the co-existence of multiple risk factors is the main cause suffering from ACS in young women; (3) the overweight cases have a trend suffering from ACS because of the combination of multiple risk factors; (4) inflammatory response is involved in the pathogenesis of ACS in young women. It may reduce the prevalence of ACS in young women to treat those risk factors.

## Supplementary information

**Additional file 1: Supplemental data table 1.** Baseline data of coronary angiographic characteristics and stents implantation in ACS group. **Supplemental data table 2.** The BMI distribution of patients in the study. **Supplemental data table 3.** Multivariate Logistic regression analysis of ACS risk factors. **Supplemental data table 4.** A bivariate correlation analysis of CRP level and age.

## Data Availability

The datasets used and/or analysed during the current study are available from the corresponding author on reasonable request.

## Abbreviations

ACS: Acute coronary syndrome
ACT: Activation coagulation time
AMI: Acute myocardial infarction
BMI: Body mass index
CHD: Coronary heart disease
CT: Computed tomography
HB: Hemoglobin
Hcy: Homocysteine
HDL: High-density lipoprotein
Hs-CRP: High sensitivity C-reactive protein
IL: Interleukin
LDL: Low-density lipoprotein
NSTEMI: Non-STEMI
PCI: Percutaneous coronary intervention
RR: Relative risk
SCr: Serum creatinine
STEMI: ST-elevation myocardial infarction
TC: Total cholesterol
TG: Triglycerides
TIMI: Thrombolysis in myocardial infarction
UA: Unstable angina
Uric: Uric acid
