# Supplementary material for: The characteristics of risk factors in Chinese young women with acute coronary syndrome
Source: BMC Cardiovasc Disord. 2020 Jun 12;20:290. doi: 10.1186/s12872-020-01577-z (PMC7291564; doi:10.1186/s12872-020-01577-z)
Supplement: Supplementary file 1 — Additional file 1: Supplemental data table 1. Baseline data of coronary angiographic characteristics and stents implantation in ACS group. Supplemental data table 2. The BMI distribution of patients in the study. Supplemental data table 3. Multivariate Logistic regression analysis of ACS risk factors. Supplemental data table 4. A bivariate correlation analysis of CRP level and age. [file 12872_2020_1577_MOESM1_ESM.pdf]

BCAR-D-20-00364

Full Title: The Characteristics of Risk Factors in Chinese Young Women with Acute Coronary Syndrome

Additional files:

Supplemental data table 1 Baseline data of coronary angiographic characteristics and stents implantation in ACS group.

Supplemental data table 2 The BMI distribution of patients in the study

Supplemental data table 3 Multivariate Logistic regression analysis of ACS risk factors

Supplemental data table 4 A bivariate correlation analysis of CRP level and age

Supplemental data table 1 Baseline data of coronary angiographic characteristics and stents implantation in ACS group.

	N	%
<40 year-old	102	24.58
40-44 year-old	313	75.42
LM	19	4.58
LAD	313	75.42
LCX	65	15.90
RCA	95	22.98
Single vessel lesion	334	80.48
Two vessel lesions	69	16.63
Three vessel lesions	12	2.98
Single stent	266	64.10
Two stents	92	22.17
Three stents	40	9.64
Four stents	10	2.41
Five stents	3	0.72
Six stents	1	0.24
Seven stents	1	0.24
Eight stents	1	0.24

Note: LM *Left main coronary artery*， LAD *Anterior descending branch of the left coronary artery*， N *Number of cases*, LCX *Left circumflex branch of the coronary artery*， RCA *Right coronary artery. Single vessel lesion*

Single vessel lesion *Single coronary artery vessel lesion,* Two vessel lesions *Two coronary artery lesions* , Three vessel lesions *three coronary artery vessel lesion.* Single stent *one stent was only implanted per patient*. Two stents *Two stents were implanted in one patients, and so on.*

In this study, all patients in the ACS group received PCI and stent implantation. The cases in the control group were normal individuals without coronary lesions. There

were no significant differences in age compared between the two groups. < 40 year-old was 102 cases (24.58%), and 40-44 year-old was 313 cases (75.42%) in the ACS group, and no significant difference compared with those in the control group (P>0.05). The characteristics of the coronary artery lesions caused ACS were LM lesions were 19 cases (4.58%), LAD lesions were 313 cases (75.42%), LCX lesions were 65 cases (15.90%), and RCA lesions were 95 cases (22.98%). Single coronary vessel lesion was 334 cases (80.48%), two-vessel lesion 69 cases (16.63%), and three- vessel lesions 12 cases (2.98%). The number of stents implantation was from one stent to eight stents per case.

Two stents referred to two stents implantation in one case, three stents mean three stent implantation for one patient, four stents mean that four stents were implanted in one case, and so on. Eight stents implantation were the most in one patient.

Supplemental data table 2. The BMI distribution of patients in the study

BMI	<24	24-26	27-29	30-39	≥40
ACS group, n(%)	150(36.14)	141(33.98)	73(17.59)	49(11.81)	2(0.48)
Control group, n(%)	346(83.37)	36(8.68)	22(5.30)	10(2.41)	1(0.24)

Note: X^2*=119.232; P=0.000.*

Body-mass index (BMI) is equal to weight (kg)/height (m^2) squared. Overweight is defined as BMI≥24. According to WHO's recommendation BMI normal range is (18.5–23.0 kg/m2) for Asians and overweight is BMI≥24. (WHO reassesses appropriate body-mass index for Asian populations. The Lancet, 2002; 360:235; WHO expert consultation. Appropriate body-mass index for Asian populations and its implications for policy and intervention strategies**.** The Lancet, 2004, 363:157-163). The Patients with a BMI greater than 24 included varying degrees of obesity. According to WHO recommendations, first degree obesity (class one obesity) is BMI 27-29, second degree (class two) obesity is BMI 30-39, and third degree (class three) obesity is BMI≥40. The BMI distribution of the patients with overweight (obesity) is shown in the following table.

The **table 2** showed that there was significant difference in the distribution of the patients with overweight (obesity) levels between the groups (P<0.01). The distribution of overweight patients (BMI≥24) in ACS group and in control group respectively 266 cases (64.10%) and 69 cases (16.63%). There were significant difference compared with two groups (X2=194.250, P=0.000).

Supplemental data table 3 Multivariate Logistic regression analysis of ACS risk factors

	B	Wald	Sig.	Exp(B)	95% C.I. for EXP(B)
Hyperlipidemia	3.176	85.436	0.000	23.940	12.209—46.943
Homocysteinemia	2.725	25.705	0.000	15.260	5.321—43.763
Overweight	2.002	95.081	0.000	7.403	4.950—11.070
High CRP	2.374	14.240	0.000	10.740	3.220—35.817
Hypertension	1.478	41.638	0.000	4.384	2.799—6.869
Hypothyroidism	2.232	13.167	0.000	9.323	2.792—31.133
Gynecological disease	1.803	17.196	0.000	6.609	2.588—14.232
Depression or anxiety	1.708	16.095	0.000	5.516	2.395—12.705
cardiac insufficiency	2.928	7.359	0.000	18.692	2.254—155.030
Hypercholesterolemia	1.665	15.193	0.000	5.287	2.228—12.214
Diabetes	1.417	17.867	0.000	4.125	2.138—7.958
Oral contraceptive	2.458	7.801	0.005	11.684	2.082—65.581
Family history of CHD	1.397	9.423	0.002	4.043	1.657—9.863
Autoimmune diseases	1.796	7.014	0.008	6.023	1.595—22.746

Note： "B" *is the statistic, that is, the correlation coefficient r；* "Wald" *is* X^2 *value of the test statistic；* "Sig." *is the P value of bilateral test；* "Exp(B)" *is the relative risk（RR）；* "95% C.I. for EXP(B)" *is the 95% confidence interval of RR；* CRP *C-reactive protein,* Family history of CHD *Family history of early onset coronary heart disease.* RR *relative risk,* 95%C.I. *95% confidence interval.*

Supplemental data table 4 A bivariate correlation analysis of CRP level and age

Correlations

		Year-old	CRP.mg/L
Year-old	Pearson Correlation	1	-.127**
	Sig. (2-tailed)		.000
	N	830	830
CRP.mg/L	Pearson Correlation	-.127**	1
	Sig. (2-tailed)	.000	
	N	830	830

Note:**. Correlation is significant at the 0.01 level (2-tailed).

CRP *C-reactive protein* ,N *Number of casese,* Sig. *is P values*
